# Antiedematogenic and Anti-Inflammatory Activity of the Monoterpene Isopulegol and Its β-Cyclodextrin (β-CD) Inclusion Complex in Animal Inflammation Models

**DOI:** 10.3390/foods9050630

**Published:** 2020-05-14

**Authors:** Andreza Guedes Barbosa Ramos, Irwin Rose Alencar de Menezes, Maria Sanádia Alexandre da Silva, Renata Torres Pessoa, Luiz Jardelino de Lacerda Neto, Fabíola Rocha Santos Passos, Henrique Douglas Melo Coutinho, Marcello Iriti, Lucindo José Quintans-Júnior

**Affiliations:** 1 Department of Biological Chemistry, Regional University of Cariri, Crato 63105-000, Brazil; andrezaurca@gmail.com (A.G.B.R.); irwinalencar@urca.br (I.R.A.d.M.); sanadiaalexandre@gmail.com (M.S.A.d.S.); trabalho.renata18@gmail.com (R.T.P.); luizjardelino@gmail.com (L.J.d.L.N.); hdmcoutinho@gmail.com (H.D.M.C.); 2 Department of Physiology, Federal University of Sergipe, São Cristóvão, Aracaju - SE 49100-000, Brazil; fabiollarsp@hotmail.com (F.R.S.P.); lucindojr@gmail.com (L.J.Q.-J.); 3 Department of Agricultural and Environmental Sciences, Milan State University, via G. Celoria 2, 20133 Milan, Italy

**Keywords:** terpenes, antiedematogenic effect, interleukin, tumor necrosis factor

## Abstract

Isopulegol (ISO) is an alcoholic monoterpene widely found in different plant species, such as Melissa officinalis, and has already been reported to have a number of pharmacological properties. Like other terpenes, ISO is a highly volatile compound that is slightly soluble in water, so its inclusion into cyclodextrins (CDs) is an interesting approach to increase its solubility and bioavailability. Thus, our aim was to evaluate the antiedematogenic and anti-inflammatory activity of isopulegol and a β-cyclodextrin–isopulegol inclusion complex (ISO/β-CD) in rodent models. For the anti-inflammatory activity evaluation, antiedematogenic plethysmometry and acute (peritonitis and pleurisy), as well as chronic (cotton pellet-induced granuloma) anti-inflammatory models, were used. The docking procedure is used to evaluate, analyze, and predict their binding mode of interaction with H1 and Cox-2 receptors. The animals (*n* = 6) were divided into groups: ISO and ISO/β-CD, negative control (saline), and positive control (indomethacin and promethazine). ISO and ISO/β-CD were able to reduce acute inflammatory activity by decreasing albumin extravasation, leukocyte migration, and MPO concentration, and reducing exudate levels of IL-1β and TNF-α. ISO and ISO/β-CD significantly inhibited edematogenic activity in carrageenan- and dextran-induced paw edema. Moreover, both significantly reduced chronic inflammatory processes, given the lower weight and protein concentration of granulomas in the foreign body granulomatous inflammation model. The results suggest that the inclusion of ISO in β-cyclodextrins improves its pharmacological properties, with the histamine and prostaglandin pathways as probable mechanisms of inhibition, and also reinforces the anti-inflammatory profile of this terpene.

## 1. Introduction

Functional foods contain ingredients that can be used for disease prevention, health promotion, and reductions of symptoms of inflammatory processes. These inflammatory processes are non-specific biological immune responses of vascular tissues that occur in response to tissue damage and various types of aggressive agents, such as pathogens, irritants, and cells with functional changes [1]. Inflammation is characterized by a series of vascular, cellular, and biochemical changes following the activation of cellular and plasma components, as well as the immune system, aiming to eliminate the aggressor and restore the integrity of the tissue [2,3]. The inflammatory response is subdivided into an acute and chronic phase, where the acute phase is short-lived, characterized by fluid and plasma element extravasation, and the migration of neutrophils into the injured tissue, induced by the release of inflammatory mediators such as TNFα, IL-1, PGE, and PAF [4]. The chronic phase occurs by the continuation of the inflammatory process, resulting in progressive changes in the cell types present at the site of the inflammatory reaction, structural changes in tissues, tissue destruction, and repair [5].

The effectiveness of anti-inflammatory drugs is assessed directly through changes in the levels of pro- and anti-inflammatory mediators, and also their ability to alter the activation state of cells involved in the inflammatory process [6,7]. The currently available anti-inflammatory drugs act by different mechanisms of action and include two main classes, glucocorticoids, and non-steroidal anti-inflammatory drugs. Glucocorticoids inhibit the synthesis of interleukins and bioactive lipids, decrease cell-mediated immunity, and leukocyte activity and number [8,9], whereas non-steroidal anti-inflammatory drugs inhibit the cyclooxygenase enzymes COX-1 and COX-2 [10,11,12]. However, long-term anti-inflammatory therapy produces a series of unwanted side effects, which has led to many studies being undertaken to try to discover new drugs that do not have this problem [6].

Cyclodextrins (CDs) are widely used in scientific research and in the pharmaceutical industry to increase the stability and solubility of drugs, and provide greater bioavailability and pharmacological effect [13,14]. CDs belong to the macrocyclic oligosaccharide family and form inclusion complexes with a wide variety of substances, changing their properties after complexation. Cyclodextrins are formed from the action of CD glycosyltransferase enzymes on starch, with the most important natural CDs being α, β, and γ [15]. Among the naturally occurring CDs, β-cyclodextrin (β-CD) is the one most commonly used for complexing drugs [14,16,17,18,19] and natural products [19,20,21,22], with the aim of potentiating their pharmacological effects through increasing their stability and solubility, and making them into promising alternative treatment options.

In terms of natural products, monoterpenes belonging to the terpene class, are a chemical group widely found in essential oils obtained from medicinal plants [23]. The monoterpene isopulegol (ISO) is found in different plant species, with several pharmacological properties being reported in the literature such as antihyperlipidemic activity [24], possible anxiolytic property [25], gastroprotective [26], analgesic [27], anticancer [28], antidiabetic [29] and an anticonvulsant activity [30] and even as a flavoring agent [31]. According to Lambe, Cadby, and Gibney [32] isopulegol is one of the flavoring substances present in several classes of food and drinks [31,33].

Thus, it was hypothesized that the inclusion of isopulegol in β-cyclodextrin would improve its pharmacological properties, making it a potential anti-inflammatory pharmacological prototype [34]. The objective of the present study was to evaluate the antiedematogenic and anti-inflammatory activity of isopulegol and its β-cyclodextrin (β-CD) inclusion complex in animal models.

## 2. Materials and Methods

The compounds used in the experiments were purchased from Sigma–Aldrich (St. Louis, MO, USA), while xylazine and ketamine were acquired from Ceva Santé Animale, BR. All substances were prepared immediately before administrations by oral, intraperitoneal, intraplantar or intrapleural routes, according to the animals’ weight (0.1 mL/10 g of body mass) and specific protocols. Isopulegol and its β-cyclodextrin complex (slurry complexation, H_2_O: 12.465 ± 0.71%; surface oil: 0.86%; total oil: 59.62%; ISOP complex: 58. 76% and complexation ratio: 1:68.3%) were provided by Dr. Lucindo Quintans of the Federal University of Sergipe [34].

### 2.1. Animals

Male and female Swiss mice (Mus musculus) weighing between 20–30 g, were obtained from the Animal Containment Unit of the Regional University of Cariri (Unidade de Contenção Animal, Universidade Regional do Cariri-URCA). The animals were housed with food and water ad libitum (Labina, Purina, Brazil) in a temperature-controlled room at 22 to 24 °C, on a 12 h light/dark cycle. Prior to the experiments, the animals were kept in the Laboratory of Pharmacology and Molecular Chemistry (Laboratório de Farmacologia e Química Molecular) of the URCA for a period of 24 h for acclimatization. The study was performed according to recommendations of the Brazilian National Council for the Control of Animal Experimentation (CONCEA) and the protocols were approved by the Experimentation Committee on the Use of Animals of the Regional University of Cariri (Comissão de Experimentação no Uso de Animais da Universidade Regional do Cariri; CEUA Nº 120/2018-GR).

### 2.2. Determination of Acute Non-clinical Toxicity of ISO and ISO/β-CD

Evaluation of the acute non-clinical toxicity was carried out according to the OECD [35], with some modifications and the table by Malone and Robichaud [36]. 

### 2.3. Evaluation of the Antiedematogenic and Anti-Inflammatory Activities

Evaluation of the anti-inflammatory activity of ISO and its inclusion complex (ISO/β-CD) was performed using paw edema models (induced by different agents): carrageenan-induced peritonitis, with vascular permeability assessments using albumin; pleurisy and the measurement of cytokines IL1-β and TNF-α; and the evaluation of chronic inflammation using the cotton pellet-induced granuloma model. The animals (*n* = 6) were divided into groups: ISO (1, 5 and 10 mg/kg), ISO/β-CD (1, 5 and 10 mg/kg) and a negative control. The histamine/prostaglandin E2 paw edema, peritonitis, pleurisy, and granuloma tests were performed with the most effective ISO and ISO/β-CD doses, this being the 10 mg/kg dose for both. The isopulegol and its inclusion complex doses were chosen based on previously published results in the literature [27].

### 2.4. Evaluation of ISO and ISO/β-CD Antiedematogenic Activity

Paw edema induced by intraplantar carrageenan/dextran administration [37,38]. Paw edema evaluation was performed according to Lapa et al. [39]. Paw edema induced by intraplantar histamine administration [40]. For the evaluation of the histamine-induced inflammatory processes, the protocol according to Maling et al. [40] was used. Paw edema induced by intraplantar prostaglandin E2 administration. Paw edema evaluation was performed according to Kawahara et al [41].

### 2.5. Edema Measurement

The plethysmometry method was used to evaluate paw edema models induced by phlogistic agents according to Winter, Risley and Nuss [37].

### 2.6. Molecular Docking 

Docking simulations were procedure Vina^®^ docking read by Chimera package. The most favorable pose, that show lowest binding free energy, aligned with binding pocket with RMSD not more than 1.0 Å was selected to analyze the interaction types using Discovery Studio 3.1 visualizer [42]. 

### 2.7. Evaluation of the Anti-inflammatory Activity of ISO and ISO/β-CD

#### 2.7.1. Peritonitis 

The carrageenan-induced peritonitis model was used to evaluate the effect of isopulegol and its inclusion complex on leukocyte recruitment according to Lapa et al. [39,43]. 

#### 2.7.2. Evaluation of Leukocyte Function and Migration, and Protein Extravasation

To evaluate protein extravasation, albumin concentrations were determined using a Labtest kit, based on the bromocresol green method which has specificity for albumin and whose absorbance is proportional to the concentration of proteins in the analyzed sample [44,45].

#### 2.7.3. Carrageenan Induced Pleurisy

The leukocyte migration was induced through the injection of carrageenan to obtain the pleural lavage according to Oliveira et al. [46]. 

### 2.8. Cytokine Measurement

The concentrations of TNF-α and IL-1β were measured using enzyme-linked immunosorbent assay (ELISA) kits (Invitrogen^®^) according to the manufacturers’ instructions. 

### 2.9. Granuloma Induced by the Implantation of Cotton Pellets

The assessment of chronic anti-inflammatory activity was performed according to Lalitha and Sethuraman [47] and total protein measurement was performed by combining the homogenate and the reagent developed by Labtest according to the manufacturers’ instructions. 

### 2.10. Statistical Analysis 

The data were analyzed by one-way ANOVA followed by Tukey’s test or two-way ANOVA followed by Tukey’s test using GraphPad Prism software (GraphPad, San Diego, CA). Values were expressed as the mean ± standard error of the mean (SEM) and differences with *p* < 0.05 being considered significant.

## 3. Results

The following results show the antiedematogenic and anti-inflammatory activities of ISO and ISO/β-CD. Then, one of the most significant results showed in this paper is that lower doses of isopulegol were shown to be effective when complexed with β-CD.

### 3.1. Acute Non-Clinical Toxicity 

Acute oral treatment with ISO and ISO/β-CD using a single oral dose of 2000 mg/kg and 5000 mg/kg did not produce any clinical signs of toxicity or death in the animals within 14 days, demonstrating a low oral toxicity. Therefore, doses <0.5% of the single 2000 mg/kg dose of ISO and ISO/β-CD (1, 5, and 10 mg/kg/v.o.) were used. Moreover, the study by Próspero et al., 2018, which evaluated the antinociceptive activity of the monoterpene isopulegol using 0.78, 1.56, 3.12, 6.25, 12.5 and 25 mg/kg doses contributed and guided the selection of the doses used in the present study.

### 3.2. Evaluation of the Antiedematogenic Activity of ISO and ISO/β-CD

#### 3.2.1. Paw Edema Induced by the Intraplantar Injection of 1% Carrageenan

Carrageenan increased edema at all assessment times, with the edematogenic peak at around the 4th hour. Oral treatment with ISO significantly reduced the edema induced by carrageenan with the doses of 5 and 10 mg/kg at all assessment times compared to the group that received only 0.9% saline, while the 1 mg/kg ISO dose only failed to show edema inhibition in the second hour of observation. ISO significantly inhibited edema at the following doses and respective evaluation times: 10 mg/kg (T1: 60%; T2: 67.74%; T3: 86.60%; T4: 84.96%); 5 mg/kg (T1: 54%; T2: 48.39%; T3: 56.70%; T4: 63.91%); 1 mg/kg (T1: 34%; T3: 43.30%; T4: 30.83%) (Figure S1-A). 

Oral administration of ISO/β-CD at the doses of 5 and 10 mg/kg significantly reduced the edema induced by carrageenan at all evaluation times, while the 1 mg/kg dose only showed significant edema inhibition at the 3- and 4-h observation times. ISO/β-CD significantly reduced edema at the following doses and respective evaluation times: 10 mg/kg (T1: 50%; T2: 59.68%; T3: 69.07%; T4: 81.20%); 5 mg/kg (T1: 36%; T2: 30.65%; T3: 45.36%; T4: 52.63%); 1 mg/kg (T3: 30.93%; T4: 30.83%) (Figure S1-B). 

It is noteworthy that the 5 and 10 mg/kg doses of ISO and ISO/β-CD showed the highest percentage of edema inhibition at the 4th hour of evaluation. Moreover, ISO/β-CD did not show a significant difference when compared to ISO at the same dose in this model, demonstrating efficacy at almost all observation times and presenting a dose-dependent effect.

#### 3.2.2. Paw Edema Induced by the Intraplantar Injection of 1% Dextran

The intraplantar injection of 1% dextran significantly increased edema, with an edematogenic peak being reached at the 2nd hour and declining in the subsequent hours. Oral administration of ISO at a dose of 10 mg/kg reduced edema at all assessment times, when compared to the saline group, with the following percentages: T1: 71.43%; T2: 85.11%; T3: 79%; T4: 75% (Figure S2-A).

ISO/β-CD at a dose of 10 mg/kg, similarly to ISO at the same dose, reduced edema at all observation times with the following percentages: T1: 85.71%; T2: 82.98%; T3: 75%; T4: 82.14%. The 5 and 1 mg/kg doses also presented edema inhibition at the 2nd hour of evaluation by 10.64% and 8.51%, respectively (Figure S2-B).

#### 3.2.3. Paw Edema Induced by the Intraplantar Injection of 1% Histamine and Prostaglandin E2

Histamine-induced edema increased significantly in the group receiving 0.9% saline, with an edematogenic peak at about 60 min after histamine administration and demonstrating a slight decline in the following hours. Promethazine (6 mg/kg/v.o.), an antihistamine used as a positive control, significantly reduced edema, when compared to the control group, demonstrating efficacy at all time intervals (T30: 78%; T60: 75%; T90: 64%; T120: 78%; T180: 88%) (Figure S3).

Treatment with ISO and ISO/β-CD at the dose of 10 mg/kg significantly reduced edema when compared to the saline group, with significance at all assessment times: ISO (T30: 89%; T60: 86%; T90: 79%; T120: 81%; T180: 92%) and ISO/β-CD (T30: 93%; T60: 96%; T90: 82%; T120: 85%; T180: 88%) (Figure S3). 

In the paw edema model resulting from PGE2 administration, treatment with ISO and ISO/β-CD at the dose of 10 mg/kg significantly reduced edema when compared to the control group at all evaluation times: ISO (T15: 80%; T30: 87%; T45: 78%; T60: 80%) and ISO/β-CD (T15: 90%; T30: 94%; T45: 84%; T60: 84%) (Figure S4).

### 3.3. Investigation of Isopulegol-Mediated COX-2 and H1 Inhibition in Silico

**Figure 1:**
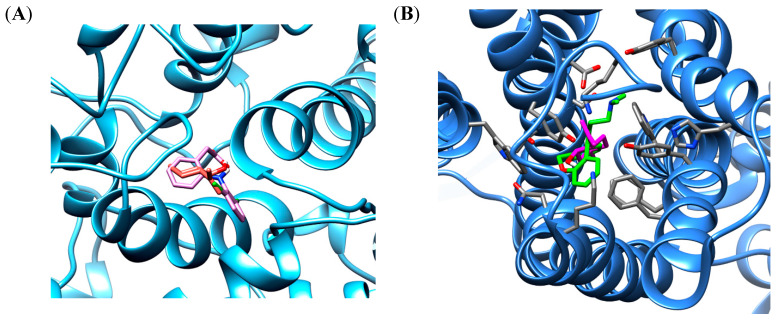
The binding poses the best stability of isopulegol (orange) and diclofenac (pink) in cyclooxygenase 2 (COX-2) binding site enzyme (**A**) and isopulegol (magenta) and doxepin (green) in binding site of H1 receptor (**B**).

The in silico docking procedure demonstrated that the root-mean-square deviations (RMSD) between the co-crystalized structure of doxepin or diclofenac and the redocked structure of ligands were 0.87 Å and 0.32 Å, respectively. This result shows a convergence in the calculated complexes formation and attesting the performance of the docking protocol. The best binding energy of isopulegol showed the same score energies of −6.4 kcal/mol to the H1 and COX-2 binding site, whereas, the docked ligands showed score energies of −8.6 kcal/mol to diclofenac and −11.5 kcal/mol for doxepin. The interaction analysis of different ligands shows hydrophobic and polar interactions in the COX-2 enzyme and H1 receptor binding sites, indicating a favorable interaction with both proteins. The best conformation of isopulegol, as well as of their respective ligands diclofenac and doxepin are shown in Figure 1. 

Docking experiments show the same favorable position for both receptors, being stabilized close to the active site by hydrophobic interactions. In the COX-2 binding site, similar cavities, with residues of VAL492, TYR354, TRP356, MET491, PHE350, LEU353, ALA496, TYR317 that form hydrophobic interactions, were observed with co-crystalized diclofenac and the non-polar part of isopulegol. There was a predominance of van der Waals, Alkyl and π-Alkyl connections in the stabilizations of the interactions. In addition, eleven similar interaction points with hydrophobic and hydrophilic cavities were found when the docked structures of isopulegol and diclofenac were compared. However, diclofenac had relatively better docking energy, in part, generated by hydrogen bonds. 

**Figure 2:**
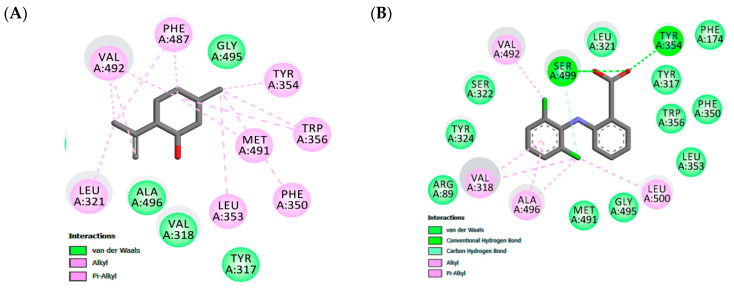

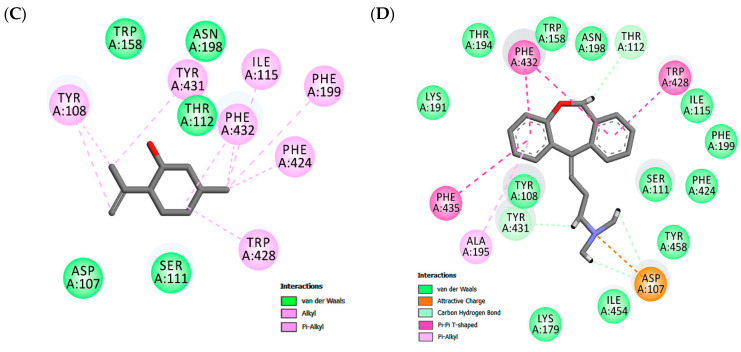
Maps of amino acid residues within binding pocket: (**A** and **C**) isopulegol, (**B**) Diclofenac; Doxepin (**D**).

In the H1 receptor binding site, isopulegol showed potential Van der Waals interactions with ASP107, SER111, THR 112, TRP158, ASN198 (Figure 2). However, other interactions such as alkyl and π-alkyl contributed to the stabilization of the interaction, as well as to the formation of the lipophilic pocket in the binding site. The GPCR family aminergic receptors are structurally similar to the H1R. The co-crystalized doxepin in the H1 receptor shows that ASP107 is a strictly conserved residue with an important interaction. However, other interactions have been reported to be essential for the binding of H1R antagonists such as doxepin in the hydrophobic pocket with residues including ILE115, PHE424, TRP428, PHE432, TRP158, and ASN198. As can be seen in Figure 2, twelve similar interaction points with hydrophobic and hydrophilic cavities were found when the docked structures of isopulegol and doxepin were compared.

Therefore, isopulegol shows favorable docking with the COX-2 enzyme and H1 receptor and, based on the similarities in anchor points, indicates that isopulegol may occupy the active site in both targets, corroborating our experimental data from the in vivo models.

### 3.4. Evaluation of the Anti-Inflammatory Activity of ISO and ISO/β-CD

#### 3.4.1. Peritonitis: Total Leukocytes, Protein Extravasation and Myeloperoxidase Measurement

The intraperitoneal administration of 1% carrageenan is a chemical stimulus capable of inducing the release of inflammatory mediators and of altering vascular permeability, causing protein extravasation from the plasma into the interstitial fluid, as well as intense leukocyte migration to the peritoneal cavity [39]. Initially, carrageenan-induced leukocyte migration was evaluated after 4 h of its administration. Figure S5 shows the inhibitory effect of ISO and ISO/β-CD (10 mg/kg) on leukocyte migration (*p* < 0.0001), as well as that of indomethacin, a non-steroidal anti-inflammatory, which significantly inhibited (*p* < 0.0001) leukocyte recruitment to the peritoneal cavity. No leukocytes were detected in the naive group, which received neither treatment nor induction.

In terms of protein extravasation, ISO and ISO/β-CD (10 mg/kg) promoted a significant reduction (*p* < 0.0001) in the quantity of albumin present in the peritoneal lavage, when compared to the group that received saline solution (Figure S6). Indomethacin, the standard drug, significantly reduced albumin extravasation (*p* < 0.0001). After 4 h of carrageenan administration into the animals’ peritoneal cavity, a significant reduction in myeloperoxidase (MPO) enzyme levels, an important marker of neutrophil infiltration, occurred in the groups treated with ISO and ISO/β-CD (10 mg/kg) compared to the group that received only saline solution (*p* < 0.001). Animals treated with indomethacin also showed a significant reduction in MPO activity (*p* < 0.001) (Figure S7).

#### 3.4.2. Pleurisy

As shown in Figure S8, injection of carrageenan into the pleural cavity of mice increased leukocyte migration as expected. Treatment with ISO 10 mg/kg, both pure and complexed, significantly reduced leukocyte migration relative to the saline group (*p* < 0.001). 

**Figure 3:**
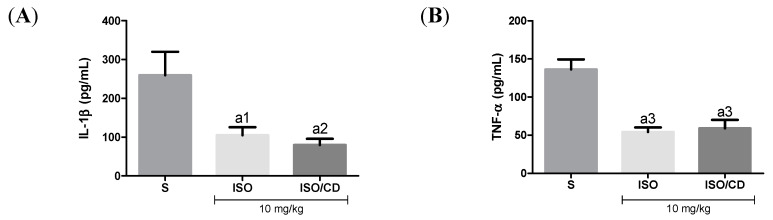
Effect of isopulegol (ISO) and β-cyclodextrin–ISO inclusion complex ISO/β-CD) (10 mg/kg/v.o.) on the inflammatory cytokines IL-1β (**A**) and TNF-α (**B**) in the pleural lavage of the animals submitted to the carrageenan-induced pleurisy. (a1-*p*<0.05; a2-*p* <0.01; a3-*p* <0.001; *vs.* saline).

In Figure 3, as expected, in mice subjected to carrageenan-induced pleurisy the levels of the pro-inflammatory cytokines TNF-α and IL-1β increased in animals treated with ISO or ISO/β-CD (10 mg/kg), IL-1β levels in the pleural lavage significantly decreased in relation to the saline group (*p* < 0.05 and *p* < 0.01, respectively), as did the TNF-α concentration (*p* < 0.001).

Effect of ISO e ISO/β-CD (10 mg/kg/v.o.) on the inflammatory cytokines IL-1β (A) and TNF-α (B) in the pleural lavage of the animals submitted to the carrageenan-induced pleurisy. (S: control/saline). Data expressed as mean ± SEM of 6 animals. Statistical analysis: One-way ANOVA followed by *Tukey’s test* with multiple comparisons. (a1 *p* < 0.05; a2 *p* < 0.01; a3 *p* < 0.001; a4 *p* < 0.0001 vs. saline).

#### 3.4.3. Effect of ISO and ISO/CD on Cotton Pellet-Induced Granulomas

The cotton pellet-induced granuloma model was used to assess the activity of ISO and ISO/β-CD in chronic inflammation [48]. Treatment with ISO and ISO/β-CD (10 mg/kg) significantly reduced the dry weight of cotton pellets (Figure S9-A) and total proteins (Figure S9-B) when compared to the group that received only saline solution, with a statistically significant correlation with the amount of granulomatous tissue, indicating that both compounds played an inhibitory role in this chronic inflammation model.

## 4. Discussion

Isopulegol, in addition to being used in the production of fragrances, has long been used as a food flavoring, being approved by the FDA (Food and Drug Administration) and the Council of Europe (1974) as an artificial flavoring substance without danger to public health. Research by the Food Chemicals Codex (1972) shows the average lethal dose (LD_50_) of isopulegol orally in rats is 1.03 ± 0.10 mL [49]. As demonstrated previously, the literature data was showed that isopolegol present several biological activity as anticancer [28], anticonvulsant activity [30], antinociceptive [27], gastroprotective effect [26], antidiabetic [29], however, there is little information about anti-inflammatory and antiedematogenic potential. In fact, the formation of inclusion complexes containing β-CD and monoterpenes has been a successful and growing approach for researchers working with essential oils and their major compounds [18].

The phlogistic agent carrageenan, a polysaccharide obtained from seaweed, and a pro-inflammatory agent widely used in well-documented models of inflammation, was used in the evaluation of the antiedematogenic activity of ISO and ISO/β-CD [50]. The inflammatory process induced by carrageenan is a complex event that involves the release of chemical mediators and cell migration into the tissues involved [37,51]. Edema development following intraplantar administration of carrageenan is a biphasic event involving mediators such as histamine, serotonin, bradykinin, and substance P in the initial phase (0–1 h). In the later phase (after 1 h), edema is maintained by the production of large amounts of prostaglandins and various cytokines such as IL-1β, IL-6, IL-10, and TNF-α [38,52].

The results showed the ISO and ISO/β-CD had an important anti-inflammatory effect at all tested doses by reducing the carrageenan-induced edema with a possible reduction in the release of inflammatory mediators. Quintans et al., (2013) found similar results in their study carried out with another monoterpene, p-cymene (PC) and its beta cyclodextrin inclusion complex (PC/CD), where both PC and PC/CD were able to markedly inhibit carrageenan-induced edema when administered orally, with the complex producing a faster and more effective response [53].

Paw edema induced by dextran administration is a model characterized by an increase in vascular permeability, kinin activation and mast cell degranulation with histamine and serotonin release, resulting in edema with a small quantity of neutrophils and proteins [38,54]. Once released, histamine and serotonin quickly act on blood vessels, triggering vasodilation with a consequent increase in blood flow to the injured site [38,54].

In studies by Silva et al. [55] and Silva et al [56], both with carvacrol, a phenolic monoterpene, reduced dextran-induced paw edema by 46% when compared to the negative control group. In other monoterpene antiedematogenic activity evaluations, 1,8-cineole (10.3; 20.6; 41.3; and 82.6 mg/kg/v.o.) reduced dextran-induced paw edema at all tested doses [57]. In the present study, the ISO and the ISO/β-CD, both at a dose of 10 mg/kg, inhibited dextran-induced edema at all evaluation times, demonstrating antiedematogenic activity against the edema caused by dextran, this being possibly the result of reduced histamine release or action on its receptors. 

Since inflammation is characterized by a series of events that include increased vascular permeability, leukocyte migration, and connective tissue proliferation, edema is one of the first signs of the inflammatory process. Histamine is a potent vasoactive mediator, whose release following mast cell activation can induce vasodilation and increase vascular permeability. Once histamine is released, production of and increased levels of prostaglandins and neuropeptides follow, leading to hyperalgesia and other pro-inflammatory events [58,59]. The study by Martins et al. [57] evaluating the monoterpene1,8-cineole in histamine-induced paw edema also showed an important antiedematogenic activity of this monoterpene, which inhibited edema by 65.95%, showing action at all evaluation times.

Prostaglandin E2 (PGE2) is considered to be a key pro-inflammatory mediator in the inflammatory process, being found at high levels in inflammatory exudates and when administered directly into tissue, and is capable of inducing a series of classic signs of inflammation [60]. PGE2 is an abundant metabolic product that plays an important role in the genesis of hyperalgesia, pyrexia, and vascular permeability, being an important pharmacological target in inflammatory disorders [61]. The antiedematogenic effect of ISO and ISO/β-CD (10 mg/kg) on the histamine and PGE2 pathway corroborates the antiedematogenic action observed in the paw edema models induced by carrageenan and dextran, which involves the participation of vasoactive amines and prostaglandins, revealing a potential action over acute phase inflammatory signs.

The binding site of the COX-2 enzyme contains two important regions of interaction formed by Tyr324, Trp356, Phe487, Phe350, Ala496, Tyr317, and Leu321 that confer a special hydrophobic characteristic in pocket and a hydrogen bond pocket formed by Ser-499 and Tyr-354 [62]. The Alkyl and Alkyl-pi stacking interactions work as “anchors”, favoring van der Waals interactions and contributing to the formation of a stable bond in the COX-2/isopulegol complex. Our results demonstrated a complementarity between the ligands and the active site of COX-2 at the same interactions, with the surrounding protein residues containing NAID-naproxen showing the hydrophobic interactions with Ala-527, Val-349, Gly-526, Trp-387, Tyr-385, and Leu-352 [63]. Previous studies with other terpene derivatives demonstrated that these compounds interacted similarly, although no involvement of hydrogen bonds was shown [64,65]. 

According to Shimamura et al. [66], taking part in the formation of the lipophilic pocket and hydrogen bond is crucial for the antagonist activity in the binding cavity of the H1 receptor. This study showed that doxepin sits deep in the ligand-binding pocket and directly interacts with TRP428 and ASP107, residues which have been reported to be essential for the binding of H1R antagonists. The tricyclic ring of doxepin that anchored in the hydrophobic pocket comprised side chains of helices III, V, and VI. Therefore, isopulegol shows favorable docking in both the target COX-2 enzyme and H1 receptor and, these results, corroborate the experimental data from the in vivo models.

Amid the biochemical changes triggered during the inflammatory process, changes in albumin levels are the most notable, with its reduced hepatic synthesis. The decrease in serum albumin levels is also associated with increased capillary permeability, one of the events in the inflammatory response, which results in albumin escaping into the extravascular space [67,68]. Inflammation induced by carrageenan involves a series of events such as leukocyte migration, plasma leakage, and the production of a variety of mediators such as cytokines, prostaglandin E2 and nitric oxide [69]. During leukocyte migration, neutrophils are the most numerous leukocytes and MPO, an enzyme stored in neutrophil granules, plays an important role as a marker of the degree of inflammation in different tissues [70].

In this study, ISO and ISO/β-CD (10 mg/kg) promoted a significant reduction in all inflammatory events induced by carrageenan. These data suggest that ISO and ISO/β-CD (10 mg/kg) can attenuate vascular events, such as vascular permeability and leukocyte migration, with a consequent reduction in MPO activity. Other monoterpenes such as bisabolol, citral, thymol and hydroxydihydrocarvone also present considerable anti-inflammatory activity, reducing vascular permeability, leukocyte migration, and myeloperoxidase activity during carrageenan-induced peritonitis [71,72,73,74].

Pleurisy is one of the classical experimental models for assessing acute anti-inflammatory activity and involves the release of a series of chemical mediators such as vasoactive amines, cytokines and prostaglandins [75,76]. The administration of carrageenan promotes a significant increase in cytokine levels, such as TNF-α and IL-1β, in the pleural exudate and propagates the inflammatory process systematically [4,77]. TNF-α and IL-1β are responsible for several characteristics of the inflammatory reaction, such as increased body temperature, neutrophil accumulation at the injury site, induction of vascular adhesion molecules and stimulation of acute phase protein synthesis [78,79,80]. Additionally, these two cytokines positively regulate the expression of cyclooxygenase 2 (COX-2) and consequently the production of prostaglandins, which are relevant in the inflammatory response [81].

Given TNF-α and IL-1β are fundamental cytokines in the inflammatory reaction, the anti-inflammatory effect of ISO and ISO/β-CD may be associated with the inhibition of cytokine release, corroborating the results presented in this study, where isopulegol and its complex (both at a dose of 10 mg/kg) inhibited the main characteristics of the inflammatory response.

In the chronic anti-inflammatory activity assessment, the cotton pellet-induced granuloma model is an important indicator of the proliferative stages of inflammation, which involves the proliferation of macrophages, neutrophils, and fibroblasts, these being fundamental elements for the formation of a granuloma [82,83,84]. During granuloma development, macrophages play a central role in the formation, maintenance and progression of a granuloma at different stages of a disease [85], with the release of inflammatory and immunomodulatory cytokines resulting from macrophage activity [86].

In this same model of chronic inflammation, the monoterpene 1,8-cineole reduced both the mass of the pellets and the total protein quantity [57], similarly to ISO and ISO/β-CD, suggesting a possible macrophage activity and fibroblast inhibition, corroborating the results from the present study, where a significant inhibition of vascular and cellular alterations such as vasodilation, vascular permeability, and cellular infiltration occurred. 

Several studies indicate that the interaction between drugs and cyclodextrins alter the pharmacokinetic properties of a drug’s active principle when complexed, altering characteristics such as solubility, stability, bioavailability, and dissolution, with beta-cyclodextrins (β-CD) being the most commonly used cyclodextrins in the drug complexation processes [87]. Da Silva Pires et al. [88], demonstrated that tenoxicam has a better dissolution profile when complexed with β-CD, resulting in a final product with greater chemical stability and better bioavailability, also reducing the adverse effects of tenoxicam.

In this study, isopulegol and its β-CD complex showed significant results against acute and chronic inflammatory models, attenuating the main responses of the inflammatory process. When compared, the results obtained by ISO and ISO/β-CD groups (10 mg/kg) did not differ statistically from each other, both showing relevant anti-inflammatory activity. However, the results obtained with ISO/β-CD (10 mg/kg) become significant, since the complexation process involves much smaller quantities of isopulegol [34], these being up to 12 times smaller in the complexed dose (10 mg/kg). Moreover, the upgrading of anti-inflammatory and modulator of pro-inflammatory cytokines profiles produced by terpenes after inclusion with CDs has been strongly described in the literature, which are corroborated by our data.

## 5. Conclusions

Together, our results demonstrated that ISO and ISO/β-CD possess antiedematogenic and anti-inflammatory activities in the animal models assessed, producing a more detailed profile of the possible benefits of this alcoholic monoterpene. Moreover, the complexation with β-CD proved to be beneficial and brought advantages that need to be highlighted, particularly as there is much less active compound in the complex compared to the pure compound, although the results are similar. The molecular docking studies indicated that the ISO mechanism of action involves the histamine and eicosanoid pathways. Thus, ISO and ISO/β-CD have shown promising anti-inflammatory properties and may be highly effective in the treatment of inflammatory disorders (acute or chronic) that cause pain. The study also provided further evidence of the possible benefits of CDs in optimizing the already appreciable pharmacological properties of terpenes.

## Supplementary Materials

The following are available online at https://www.mdpi.com/2304-8158/9/5/630/s1, ***Figure S1***: Effect of ISO and ISO/β-CD (1, 5 and 10 mg/kg/v.o.) on paw edema induced by intraplantar injection of 1% carrageenan in mice; ***Figure S2***: Effect of ISO and ISO/β-CD (1, 5 and 10 mg/kg/v.o.) on paw edema induced by intraplantar injection of 1% dextran in mice; ***Figure S3***: Effect of ISO and ISO/β-CD (1, 5 and 10 mg/kg/v.o.) on paw edema induced by intraplantar injection of 1% histamine in mice; ***Figure S4***: Effect of ISO and ISO/β-CD (1, 5 and 10 mg/kg/v.o.) on paw edema induced by intraplantar injection of prostaglandin E2 in mice; ***Figure S5***: Effect of ISO and ISO/β-CD (10 mg/kg/v.o.) on leukocyte migration in 1% carrageenan-induced peritonitis; ***Figure S6***: Effect of ISO and ISO/β-CD (10 mg/kg/v.o.) on protein extravasation in 1% carrageenan-induced peritonitis; ***Figure S7***: Effect of ISO and ISO/β-CD (10 mg/kg/v.o.) on MPO activity in 1% carrageenan-induced peritonitis; ***Figure S8***: Effect of ISO and ISO/β-CD (10 mg/kg/v.o.) in the leukocytes migration in mice submitted to carrageenan-induced pleurisy; ***Figure S9***: Effect of ISO and ISO/β-CD (10 mg/kg/v.o.) on granuloma formation.
