# Supplementary material for: Antiedematogenic and Anti-Inflammatory Activity of the Monoterpene Isopulegol and Its β-Cyclodextrin (β-CD) Inclusion Complex in Animal Inflammation Models
Source: Foods. 2020 May 14;9(5):630. doi: 10.3390/foods9050630 (PMC7278878; doi:10.3390/foods9050630)
Supplement: Supplementary file 1 [file foods-09-00630-s001.pdf]

All Figures

Figure A1 - Effect of ISO and ISO/β-CD (1, 5 and 10 mg/kg/v.o.) on paw edema induced by intraplantar injection of 1% carrageenan in mice.

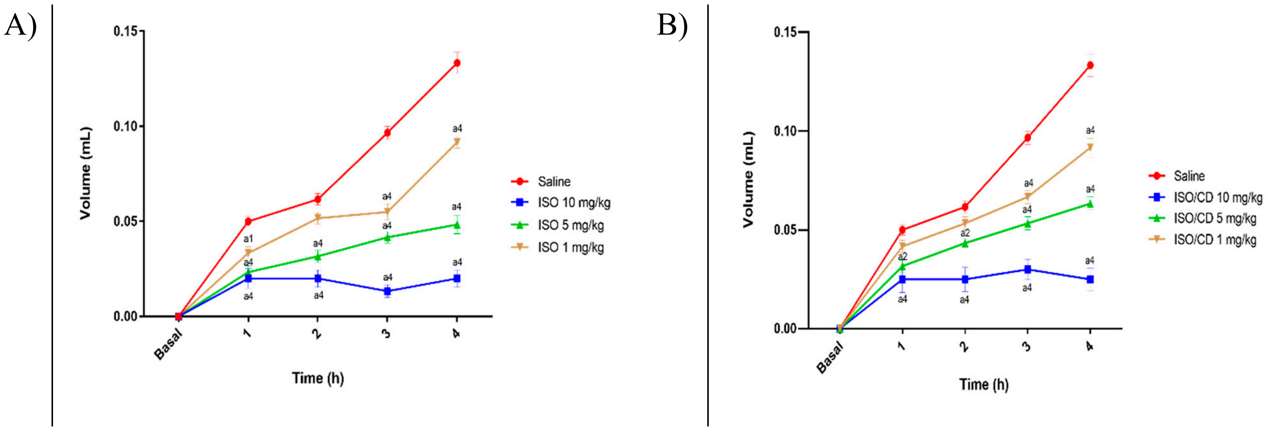

Effect of ISO (A) and ISO/β-CD (1, 5 and 10 mg/kg/v.o.) (B) on paw edema induced by the intraplantar injection of 1% carrageenan in mice up to the 4th hour. Values represent the mean ± S.E.M. (n=6/group). Statistical analysis: Two-way ANOVA followed by Tukey's Test with multiple comparisons (a1*p*<0.05; a2*p* <0.01; a3*p* <0.001; a4*p* <0.0001 *vs.* saline).

Figure A2 - Effect of ISO and ISO/β-CD (1, 5 and 10 mg/kg/v.o.) on paw edema induced by intraplantar injection of 1% dextran in mice.

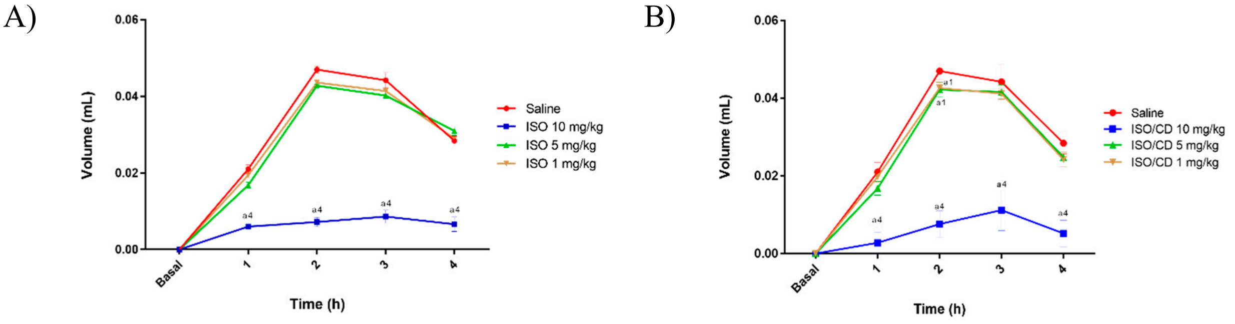

Effect of ISO (A) and ISO/β-CD (1, 5 and 10 mg/kg/v.o.) (B) on paw edema induced by the intraplantar injection of 1% dextran in mice up to the 4th hour. Values represent the mean ± S.E.M. (n=6/group). Statistical analysis: Two-way ANOVA followed by Tukey's Test with multiple comparisons (a1*p*<0.05; a2*p* <0.01; a3*p* <0.001; a4*p* <0.0001 *vs.* saline)

Figure A3 - Effect of ISO and ISO/β-CD (1, 5 and 10 mg/kg/v.o.) on paw edema induced by intraplantar injection of 1% histamine in mice.

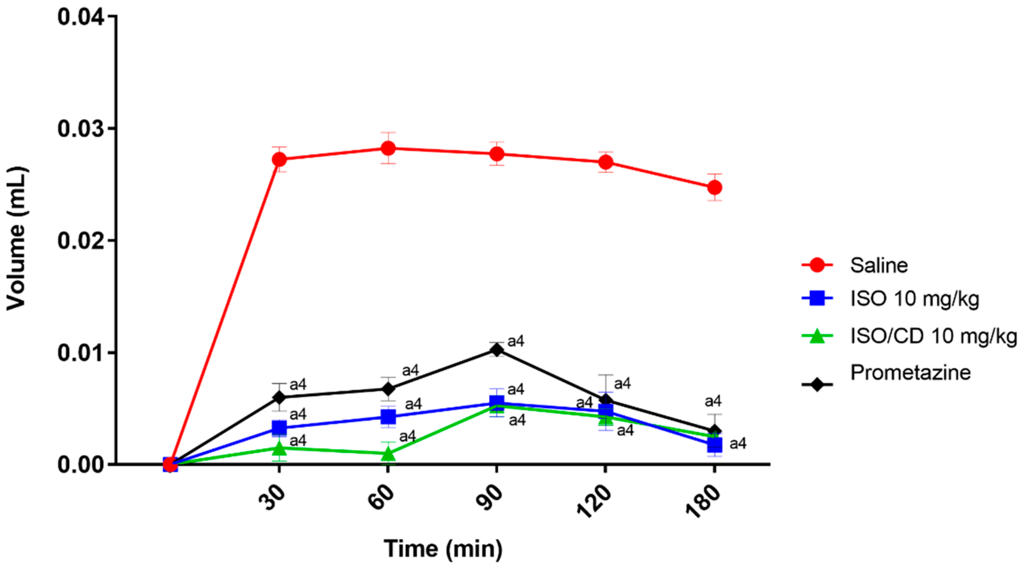

Effect of ISO (A) and ISO/β-CD (1, 5 and 10 mg/kg/v.o.) (B) on paw edema induced by the intraplantar injection of 1% histamine in mice up to 180 minutes. Values represent the mean ± S.E.M. (n=6/group). Statistical analysis: Two-way ANOVA followed by Tukey's Test with multiple comparisons (a1*p*<0.05; a2*p* <0.01; a3*p* <0.001; a4*p* <0.0001 *vs.* saline).

Figure A4 - Effect of ISO and ISO/β-CD (1, 5 and 10 mg/kg/v.o.) on paw edema induced by intraplantar injection of prostaglandin E2 in mice.

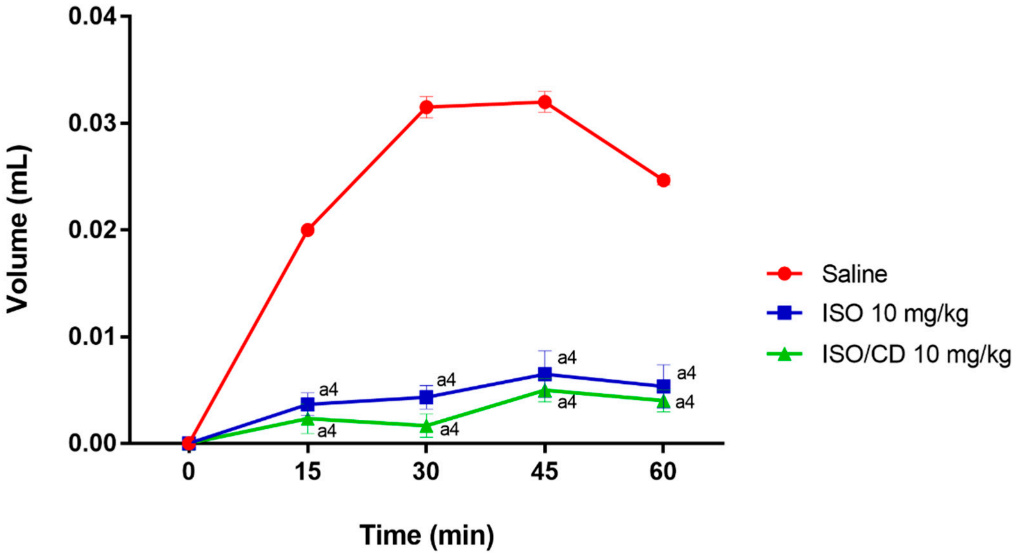

Effect of ISO (A) and ISO/β-CD (1, 5 and 10 mg/kg/v.o.) (B) on paw edema induced by the intraplantar injection of PGE2 in mice up to 60 minutes. Values represent the mean ± S.E.M. (n=6/group). Statistical analysis: Two-way ANOVA followed by Tukey's Test with multiple comparisons (a1*p*<0.05; a2*p* <0.01; a3*p* <0.001; a4*p* <0.0001 *vs.* saline).

Figure A5 - Effect of ISO and ISO/β-CD (10 mg/kg/v.o.) on leukocyte migration in 1% carrageenan-induced peritonitis.

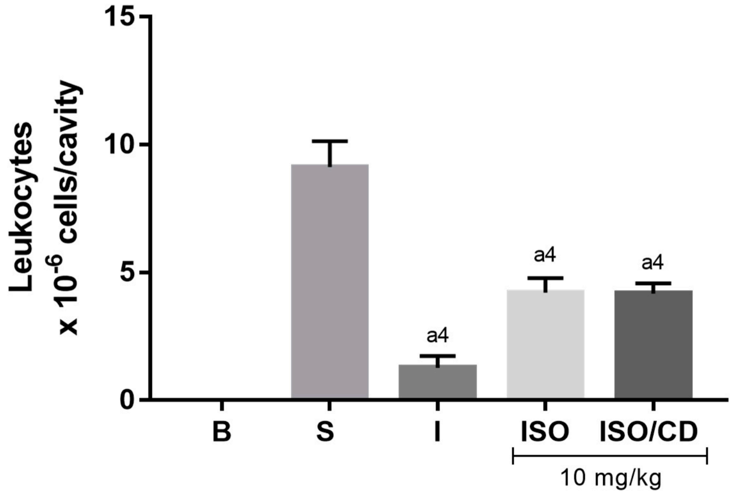

Effect of ISO and ISO/β-CD (10 mg/kg/v.o.) on leukocyte migration in peritonitis induced by 1% carrageenan in mice (B: blank; S: control/saline; I: indomethacin 25 mg/kg). Values represent the mean ± S.E.M (n=6/group). One-way ANOVA followed b y Tukey's Test with multiple comparisons (a1p<0.05; a2p <0.01; a3p <0.001; a4p <0.0001 *vs.* saline).

Figure A6 - Effect of ISO and ISO/β-CD (10 mg/kg/v.o.) on protein extravasation in 1% carrageenan-induced peritonitis.

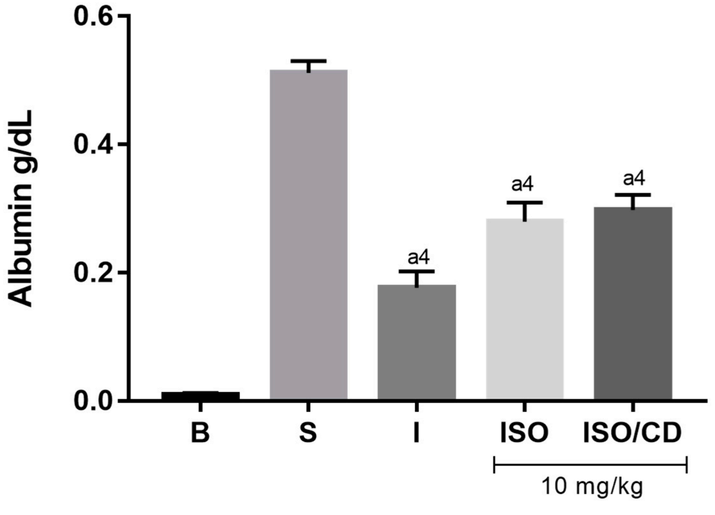

Effect of ISO and ISO/β-CD (10 mg/kg/v.o.) on protein extravasation in peritonitis induced by 1% carrageenan in mice (B: blank; S: control/saline; I: indomethacin 25 mg/kg). Values represent the mean ±

S.E.M (n=6/group). One-way ANOVA followed by Tukey's Test with multiple comparisons (a1*p*<0.05; a2*p* <0.01; a3*p* <0.001; a4*p* <0.0001 *vs.* saline)

Figure A7 - Effect of ISO and ISO/β-CD (10 mg/kg/v.o.) on MPO activity in 1% carrageenan-induced peritonitis.

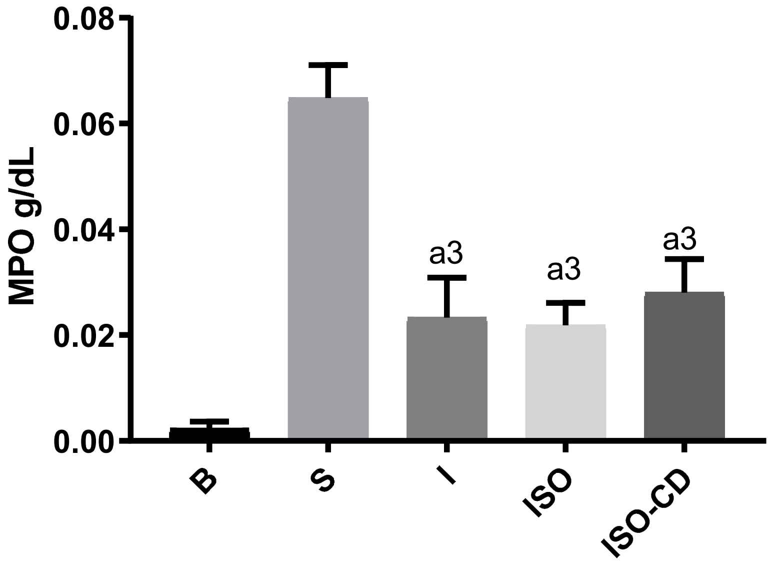

Effect of ISO and ISO/β-CD (10 mg/kg/v.o.) on MPO activity in peritonitis induced by 1% carrageenan in mice (B: blank; S: control/saline; I: indomethacin 25 mg/kg). Values represent the mean ± S.E.M (n=6/group). One-way ANOVA followed by Tukey's Test with multiple comparisons (a1*p*<0.05; a2*p* <0.01; a3*p* <0.001; a4*p* <0.0001 *vs.* saline).

Figure A8 - Effect of ISO and ISO/β-CD (10 mg/kg/v.o.) in the leukocytes migration in mice submitted to carrageenan-induced pleurisy.

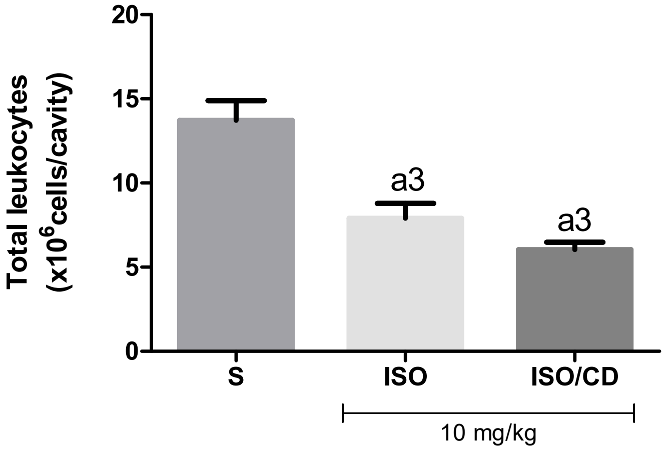

Effect of ISO e ISO/β-CD (10 mg/kg/v.o.) in the leukocytes migration in mice submitted to carrageenan-induced pleurisy. (S: control/saline). Data were expressed as mean ± SEM of 6 animals. Statistical analysis: One-way ANOVA followed by *Tukey's test* with multiple comparisions. (a1p<0,05; a2p<0,01; a3p<0,001; a4p<0,0001 vs. saline).

Figure A9 - Effect of ISO and ISO/β-CD (10 mg/kg/v.o.) on granuloma formation.

A) B)

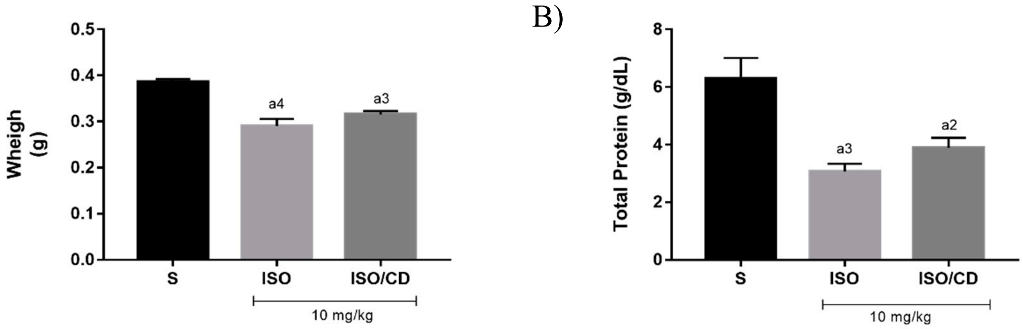

Effect of ISO and ISO/β-CD (10 mg/kg/v.o.) on granuloma formation. (S: control/saline). A) Granuloma weight; B) Total proteins obtained from the granuloma homogenate. Values represent the mean ± S.E.M (n=6/group). One-way ANOVA followed by Tukey's Test with multiple comparisons (a1*p*<0.05; a2*p* <0.01; a3*p* <0.001; a4*p* <0.0001 *vs.* saline).
